# Determination of Viscosity and Surface Tension for High LiF and KF Content Electrolytes with Innovative Testing Method in Aluminum Electrolysis

**DOI:** 10.3390/ma19081587

**Published:** 2026-04-15

**Authors:** Bo Hong, Benjun Xu, Tao Yang

**Affiliations:** 1 Guiyang Aluminum and Magnesium Design and Research Institute Co., Ltd., Guiyang 550001, China; 13511168185@163.com; 2 Materials and Metallurgy College, Guizhou University, Guiyang 550002, China; bjxu@gzu.edu.cn

**Keywords:** viscosity, surface tension, aluminum electrolyte

## Abstract

This trend is inevitable when aluminum electrolyte raw materials containing a higher content of LiF and KF are being widely used. Though these substances are often used as regulators in aluminum electrolysis due to their high corrosivity, measuring the physical parameters of the electrolyte through ordinary measurement methods is difficult. In this paper, electrolytes with different Li and K contents at different temperatures were synthesized as simulation samples, and viscosity and surface tension were measured by a new method. The results showed that in the aluminum electrolyte with a cryolite ratio of three, KF and LiF had similar impacts on viscosity when the mechanisms were completely different. As the content increased, KF reduced the viscosity of the material through the ion lattice spacing effect, and the high mobility of LiF contributed greatly to the viscosity reduction. However, the viscosity reduced significantly only when there was a higher KF concentration. The effects of the two regulators on the surface tension were different. The rising LiF content presented a greater effect on the surface tension, and the temperature was also a main factor affecting the surface tension of the electrolyte. When the two were added together in the electrolyte, a high content of KF helped reduce the surface tension. The research in this paper plays a role in promoting the exploration of phase parameters of aluminum electrolytes with high Li and K content.

## 1. Introduction

Aluminum electrolysis plays a significant role in the country’s economic construction as the cornerstone of the national industrial system [1]. In actual aluminum electrolysis production, high LiF and KF content electrolytes are widely used as raw materials [2]. Adding these additional fluorides is also the main method for adjusting the electrolytic conductivity in the traditional process. However, without a systematic study, these conditional changes will inevitably lead to changes in other parameters. Firstly, the phase of solid electrolytes will increase while the electrolytes’ XRD accuracy declines due to high LiF and KF content, etc. [3]. Compared with the previous materials, these conditions lead to an initial electrolyte crystal temperature decrease in varying degrees, which disrupts the thermal balance of the electrolytic bath [4]. The effect on other parameters can also be different for LiF and KF additives. On the one hand, rising LiF content may enhance the electrical conductivity, while rising KF content can be opposite to it [5]. On the other hand, during the electrolysis process, KF will increase the carbon slag content and deteriorate the solubility performance. Therefore, although Li and K enrichment may enhance electrical conductivity, it is offset by indirect effects such as carbon slag retention, solubility decline, and thermal equilibrium disruption, which causes production indicator deterioration [6]. To avoid these situations, it is necessary to explore the influence of high Li/K enrichment on the electrolyte physical properties.

Two significant physical properties will be explored as follows: surface tension and viscosity. Surface tension plays a decisive role in the stability of the melting interface [7]. The surface tension helps to improve the current efficiency and reduce the dissolution loss of aluminum [8]. A higher value of surface tension also contributes to the precipitation of anode gas. Viscosity has an impact on the movement of small aluminum beads in the electrolytic bath and the sedimentation of solid alumina particles [9]. However, excessive viscosity will hinder the overflow of anode gas, which reduces the efficiency of mass transfer and heat transfer [10]. Therefore, using materials with high surface tension and moderate viscosity is more conducive to aluminum electrolysis.

The conventional device for testing viscosity in the laboratory is a rotational rheometer [11]. Its principle is that the motor drives the rotating shaft to rotate to overcome the viscous resistance of the liquid, and calculates the viscosity according to the flow law of Newtonian fluids [12]. Due to the low viscosity of the aluminum electrolyte with high enrichment of Li and K and the corrosiveness of the fluoride salts in this electrolyte to the rotor, it is often difficult to measure [13]. To address this, we have made improvements by changing the material of the rotor and increasing its length, enabling more accurate measurement of data under corrosion-resistant conditions.

In conclusion, we have explored the influence of Li and K content on two-phase parameters by altering the content of Li and K in the electrolyte to change the viscosity and surface tension of the electrolyte at different temperatures, and thereby determine the optimal Li and K content in the electrolyte.

## 2. Materials and Methods

### Materials

**Figure 1:**
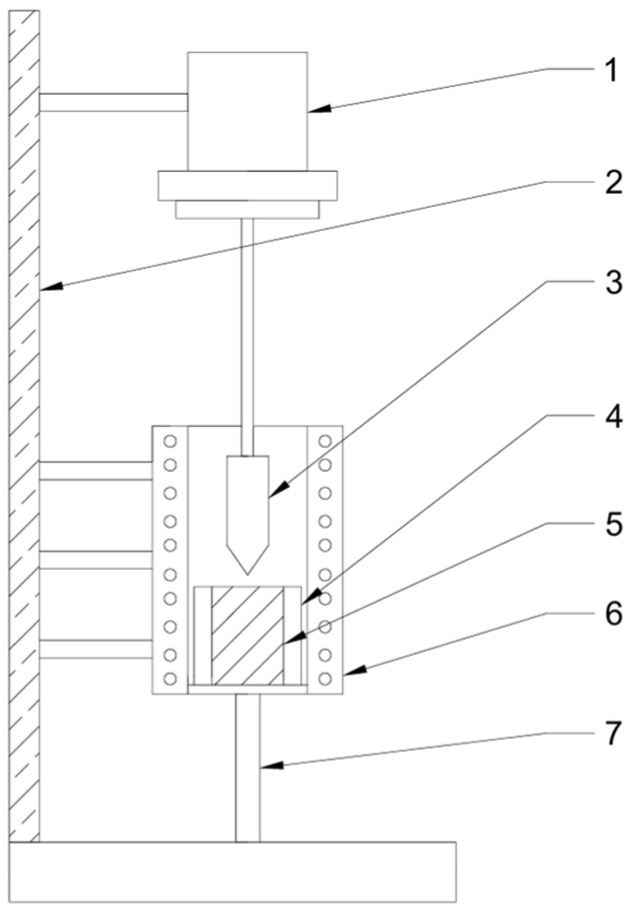
The structure of the viscosity testing device: 1. probe system DSR502, 2. elevating mechanism, 3. graphite probe, 4. inner core, 5. melt samples, 6. shaft resistance furnace, and 7. adamantine spar.

NaAlF_3_ was purchased from Shandong Xiya Chemical Co., Linyi, China. Al_2_O_3_, LiF, and KF were all purchased from Shanghai Macklin Biochemical Co., Shanghai, China. The simulated electrolyte was composed of sodium fluor aluminate, alumina, lithium fluoride, and potassium fluoride in different proportions. The structure of the viscosity testing device is shown in Figure 1. First, sodium fluoride and alumina were weighed by a precision balance, and their cryolite ratio was 3. After grinding for 30 min, the samples were prepared, and the blank control group of the electrolyte was simulated. Different molar ratios of LiF (1%, 3%, 5%, 7%) and KF (1%, 3%, 5%, 7%) were added as simulated electrolytes for high enrichment of lithium and potassium while keeping the cryolite ratio of the blank control group unchanged.

The simulated electrolyte or blank control sample with high enrichment of Li and K was put into a modified rotary viscometer for measurement. Firstly, the sample was loaded into a graphite crucible and placed in a vacuum drying oven at 100 °C to dry the moisture, and then the crucible was placed in a well-type electric furnace, and the temperature was slowly heated to the sample melting point, and the sample temperature was measured. The heated crucible is transferred to the inner cylinder of the viscometer container, and the micro-viscosity test device (probe system DSR502, Guizhou university, Guiyang, China) is vertically placed into the inner cylinder through the lifting mechanism to test the electrolyte viscosity at this temperature.

The calculation formula of viscosity is as follows:

(1) $$\tau =\eta \frac{du}{dy}$$

where τ is the shearing stress, Pa, η is viscosity, Pa/s, and du/dy is the velocity gradient, s^−1^.

The molten electrolyte is a Newtonian fluid, and the relationship between viscosity and temperature conforms to the Arrhenius formula. The formula for viscosity and temperature is as follows:

(2) $$\ln(\eta )=\ln(A)+\frac{Ea}{R}\times \frac{1}{T}$$

where η is viscosity, Pa/s; A is the pre-exponential factor related to molecular shape; Ea is the shear activation energy, J/mol; R is ideal gas constant, J/(mol·K); and T is kelvin temperature, K.

The surface tension testing was conducted by the same device that was used in viscosity testing. The only thing changed was the probe. The formula for the surface tension is as follows:

(3) $$\gamma =\frac{W}{\Delta A}$$

where γ is surface tension, N/m; W is the power provided by mechanics, J, ∆; and A is the changed surface area, m^2^.

The in situ crystalline structures of the samples were characterized by using a powder X-ray diffraction technique (XRD, X’Pert Pro MPD, PANalytical B.V., Almelo, The Netherlands) with continually rising temperature from 900 to 950 °C. The diffraction meter was equipped with a copper target Kα, the scanning rate was 5°/min, and the scanning range was 10–90°.

## 3. Results and Discussion

### 3.1. Cryolite Ratio

Cryolite ratio is one of the core control parameters in the aluminum electrolysis process, directly affecting the physical and chemical properties and metallurgical chemical behavior of the electrolyte [14]. Precise control of the cryolite ratio can significantly lower the electrolysis temperature, thereby reducing energy consumption, decreasing fluorine emissions, and enhancing current efficiency [15]. It is necessary to balance its negative impact on the solubility/dissolution rate of alumina and the conductivity of the electrolyte.

For the determination of the cryolite ratio, physical and chemical property tests were conducted on the collected samples from the local aluminum industry corporation. This investigated the influence of these properties on the cryolite ratio. Measuring the surface tension and viscosity of these samples helped to determine the appropriate cryolite ratio in further experiments.

**Figure 2:**
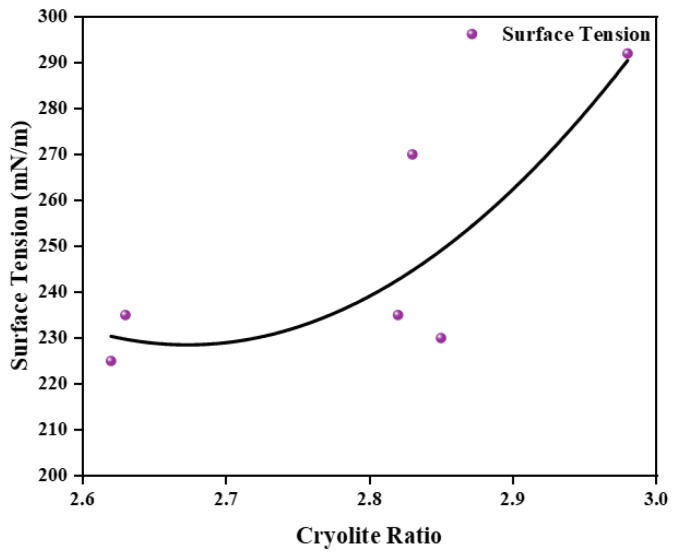
The fitting curve of surface tension and cryolite ratio.

Based on the test results of the received samples, the simulation curve is shown in Figure 2 to explain the relationship between the surface tension and cryolite ratio. As the cryolite ratio increased, the surface tension decreased. It increased after the inflection point, which was at 2.70. According to this curve, it can be inferred that the surface tension of electrolytes with a cryolite ratio of three was greater than that of electrolytes with a cryolite ration less than three. This phenomenon met the previous requirement, which was that high levels of Li and K electrolytes need high surface tension.

**Figure 3:**
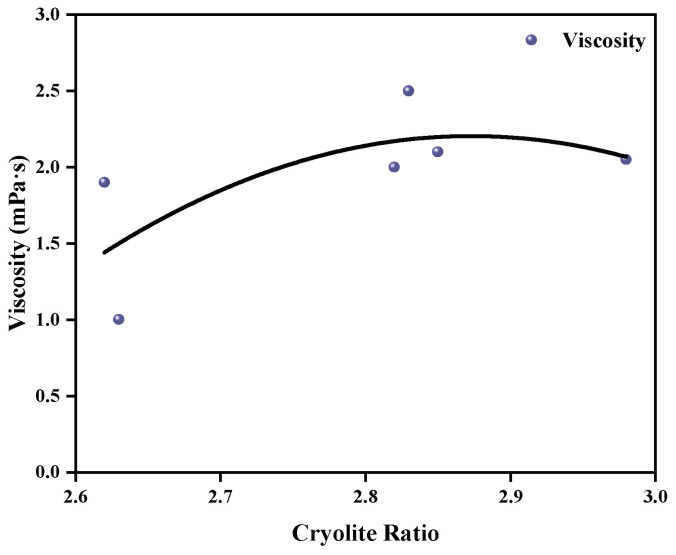
The fitting curve of viscosity and cryolite ratio.

Similarly, viscosity tests were conducted on the same samples, and the fitting curve was obtained. The results are shown in Figure 3. The viscosity of the samples was mostly maintained between two and three. As the cryolite ratio increased from 2.65 to 2.95, the viscosity of the samples first increased and then gradually decreased. The maximum viscosity value was 2.2 mPa·s at 2.825. According to this curve, it can be deduced that when the cryolite ratio reaches three, the viscosity is slightly lower than 2 mPa·s. These results meet the requirements of electrolytes enriched with high levels of Li and K. In conclusion, the electrolytes with a cryolite ratio of three have met the expectation of high surface tension and moderate viscosity after general investigation. Therefore, a cryolite ratio of three was considered an appropriate condition for subsequent experiments.

### 3.2. Experiments for Viscosity Testing

**Figure 4:**
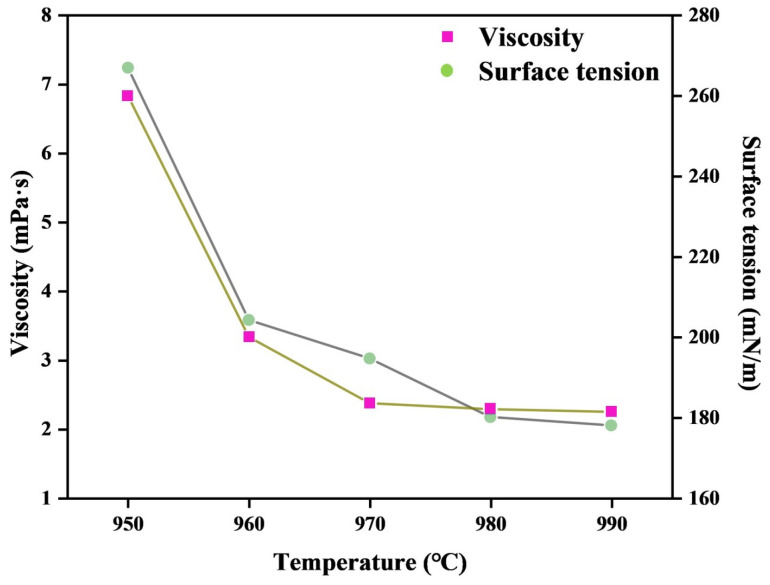
The surface tension and viscosity of the electrolyte without LiF and KF with a cryolite ratio of three at different temperatures.

Following the determination of the electrolyte cryolite ratio as three, a reference sample consisting of 97 wt% sodium fluoroaluminate and 3 wt% alumina was prepared to establish baseline properties. The surface tension and viscosity of this reference electrolyte were measured across a range of temperatures. Results of Figure 4 indicated that both surface tension and viscosity gradually decreased with increasing temperature. This trend could be attributed to the enhanced thermal motion of ions or molecules within the melt at higher temperatures, which weakened the intermolecular interactions. For the pure electrolyte system, complete melting was not achieved below 950 °C, a finding that aligned with actual industrial practice. It was also noted that the introduction of Li and K into the electrolyte lowered its melting point and consequently altered its viscosity and surface tension. Further investigation was focused on the effects of these compositional modifications on the physicochemical properties of the electrolyte.

**Figure 5:**
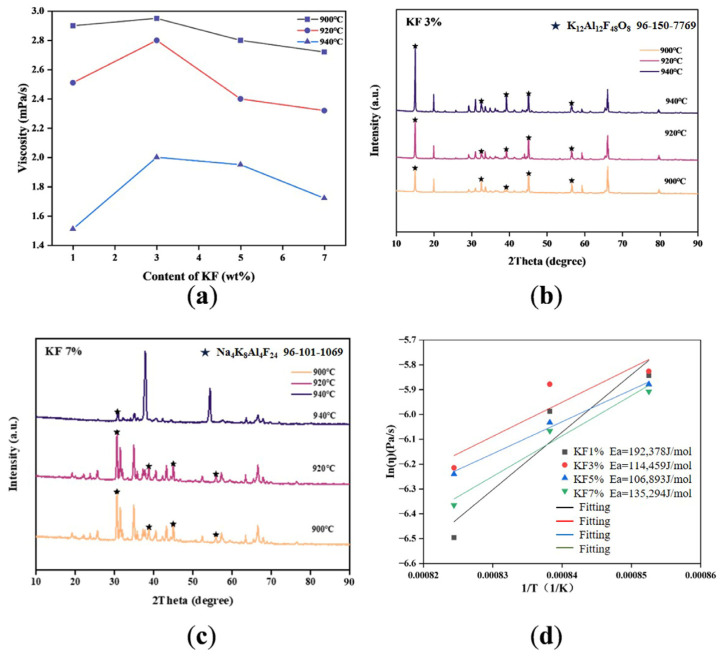
(**a**) Viscosity of electrolytes at different temperatures with different KF content. (**b**) XRD images of 3% KF. (**c**) XRD images of 7% KF. (**d**) Shear activation energy of electrolytes at different temperatures of different KF contents.

After the cryolite ratio of the simulated electrolyte was determined to be three, potassium fluoride with different molar ratios (1, 3, 5, and 7) was added to the electrolytes. These were tested at different temperatures (900, 920, and 940 °C) to obtain their viscosity. The results showed that the electrolyte viscosity for those containing only KF was the highest at 900 °C, and the viscosity gradually decreased with the temperature increase. It is speculated that the increase in temperature gave higher kinetic energy to molecules and ions, and the intermolecular force of the electrolyte gradually decreases [16]. At the same temperature, with the increase in KF content, the viscosity increased first and then decreased, reaching the highest at 3 wt%. The shear activation energy calculated in Figure 5 also depicted that with the increase in KF content, the shear activation energy generally showed a downward trend when the KF content was less than 2 wt%. This phenomenon can be attributed to the fact that the radius of the potassium ion is larger (138 pm), which disturbed the originally dominant ion (such as Na^+^) network after entering the melt [17]. In the early stage, a small amount of K^+^ was embedded in the complex ion gap, such as [AlF_6_]^3−^. Due to the change in steric hindrance and electrostatic interaction, local ion migration was blocked, which showed a temporary increase in viscosity. Also, a smaller amount of K^+^ may be involved in the formation of the K-Al-F transitional complex structure. The images of XRD gave exact proof of the existence of the K-Al-F structure. The diffraction peaks of KALOF and NaKAlF phases were shown clearly in XRD images at different temperatures and different KF contents, respectively. As the temperature rose, the crystal phase of the K-Al-F structure could not be detected in 7% KF content, and it can be speculated that the structure may be destroyed. These structures also showed less stability and larger volume in the researched literature, which temporarily increased the internal friction resistance of the melt [18]. By adding more K^+^ ions, the K-Al-F structure of the molten salt internal system was destroyed, which led to the viscosity reduction. This phenomenon is similar to the change rule of high coordination groups in molten salt, indicating that the viscosity of molten salt is directly affected by the change in coordination groups in the system [19].

**Figure 6:**
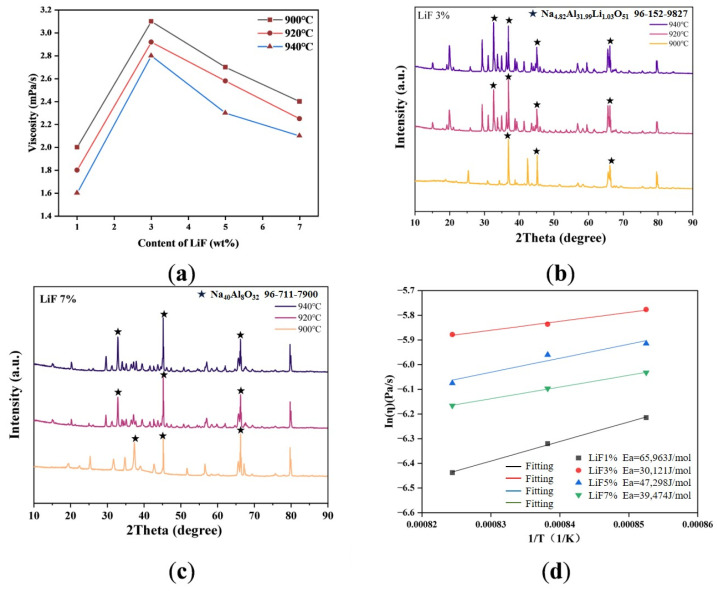
(**a**) Viscosity of electrolytes in different temperatures with different LiF content. (**b**) XRD images of 3% LiF. (**c**) XRD images of 7% LiF. (**d**) Shear activation energy of electrolytes at different temperatures of different LiF contents.

In the same case, a similar change rule was demonstrated for the viscosity of the electrolyte when LiF was present. As the temperature increased, the viscosity decreased. The viscosity increased first and then decreased at the same temperature, and 3 wt% is the inflection point of the curve. Different from KF, the viscosity changes of the electrolyte showed strong regularity, and the slope of the curve was large. From the fitting curve in Figure 6, it can also be seen that with the increase in LiF content, the shear activation energy showed a decreased trend too. This may suggest that the initial addition of LiF to occupy the pores made the viscosity increase; however, the small ionic radius of lithium ions can break the anions with a high degree of polymerization ([AlF_6_]^3−^) in the cryolite melt due to the high charge density of increasing LiF content, which promotes its depolymerization into a smaller unit ([AlF_4_]^−^) [20]. This process directly reduces the average ion size of the melt and the flow resistance, and thus regularly reduces the viscosity. It has also been proven in the literature that LiF has a certain effect on reducing viscosity. The disappearance of NaLiALO diffraction peaks and the appearance of NaAlO in the XRD image also proved that Li^+^ changed into an ionic state in the melt. Due to the strong conductivity of Li^+^, the electric mobility increased. This led to the free volume caused by Li^+^ in the melt, and the disturbance to the melt structure was smaller, and the migration resistance was lower, which gave it a certain lubrication effect and further reduced the viscosity [21].

**Figure 7:**
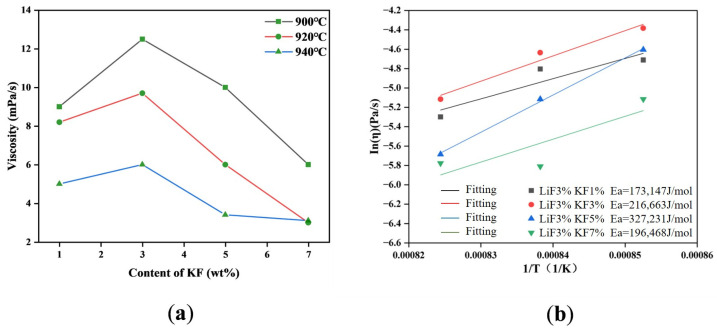
(**a**) Viscosity of electrolytes at different temperatures with different KF content when the LiF content was 3 wt%. (**b**) Shear activation energy of electrolytes at different temperatures of different KF contents when the LiF content was 3 wt%.

When the lithium fluoride content is fixed at 3 wt% with temperature change, the trend of viscosity with potassium fluoride content is shown in Figure 7. Similarly, as the temperature increased, the viscosity decreased, and then viscosity increased first and then decreased at the same temperature, and 3wt% is the inflection point of the curve. It can also be seen from the fitting curve in Figure 7 that under the combined effect of KF and LiF, the shear activation energy was more affected by KF, and only when the KF content was up to 5%, the shear activation energy decreased. This demonstrated that the presence of LiF and KF together affected the viscosity of the electrolyte analogously to the two existing alone. However, compared with a single component in the electrolyte, the viscosity increased greatly with a small amount of KF, which may be due to the ionic size effect of K^+^. At the same time, LiF promoted the conductivity of the electrolyte, while KF was the opposite. This result also showed that the conductivity and lubrication of LiF were greatly affected by the existence of a small content of KF. Then, continuing to increase KF, viscosity decreased. This phenomenon may be attributed to the fact that excessive KF changed the original system; the free volume between the large ions (K^+^) got larger, and the average distance between the ions increased, resulting in the electrostatic attraction becoming weaker, making the ion clusters easier to slide, so that the viscosity decreased. Furthermore, adding KF content to excess also meant adding F^−^ ions, and the [AlF_6_]^3−^ in the electrolyte was more likely to change to the [AlF_4_]^−^ with a smaller ionic radius. Thus, the main composition of the volume in the system, which was dominated by the larger radius anion ([AlF_6_]^3−^), transformed to one that was dominated by the larger radius cation (K^+^) and the smaller radius anion ([AlF_4_]^−^, F^−^). The following properties of [AlF_4_]^−^, such as less charge, smaller ionic resistance, and stronger mobility, finally led to the decrease in electrolyte viscosity with high KF content.

### 3.3. Experiments for Surface Tension Testing

**Figure 8:**
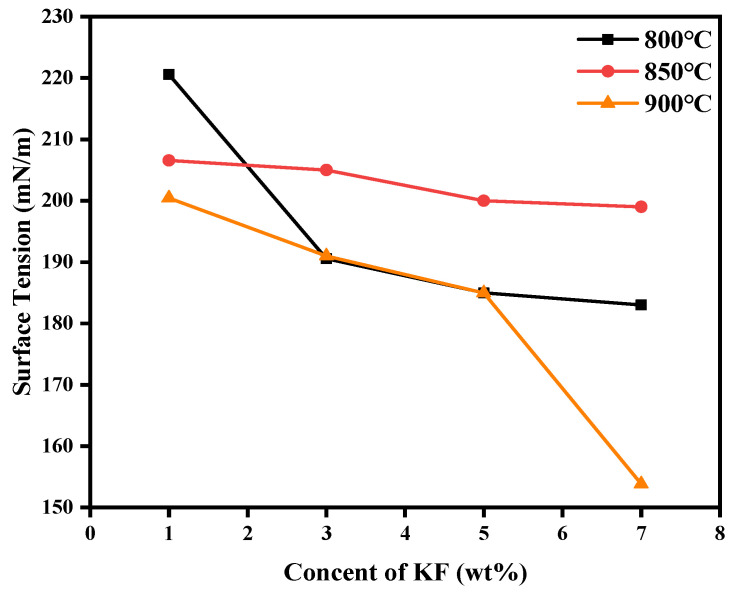
Surface tension of electrolytes at different temperatures with different KF content.

When measuring the surface tension of the electrolyte, first of all, the sample without LiF and KF melted completely, and its surface tension value was 168.954 mN/m at 940 °C. At this time, the surface tension of the electrolyte did not change significantly in a small temperature range (800–900 °C). In the literature we have reviewed, the addition of KF reduced the surface tension of the electrolyte. Therefore, the reduced temperature and increased temperature gradient were settled to explore the law between the surface tension and the electrolyte after adding KF at different temperatures. The results are shown in the Figure 8. At the same temperature, as expected, the surface tension decreased with the increase in KF content. At 850 °C, the electrolyte maintained a high surface tension and changed slowly. At other temperatures, only the electrolyte containing excessive KF content (7 wt%) reduced the surface tension of the electrolyte below the sample without LiF and KF. This kind of phenomenon may be attributed to the following: At 850 °C, KF lost its function as a surfactant when CR equaled 3 at 850 °C. K^+^ cannot be adsorbed by the melt surface effectively, so the surface tension was not sensitive to the change in KF concentration. At 800 °C, the melt structure may be more rigid, and the ion mobility is lower. Due to the difference in the interaction energy between main melt particles (Na^+^, AlF_3_^−^) and the added K^+^ ion, the added K^+^ ion is squeezed to the surface, thereby reducing the surface tension [22]. As the temperature increased to 900 °C, the thermal motion of the melt was intense [23]. The kinetic factor was the dominant influence: the K^+^ ions obtained enough energy to overcome the energy barrier, and the process of migration and adsorption to the surface became easier. Thus, the surface activity effect of KF reemerged [24].

**Figure 9:**
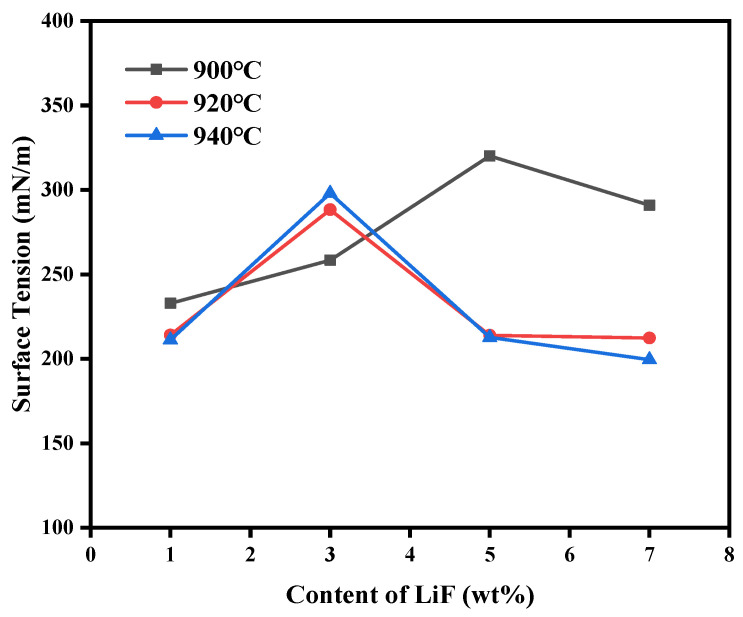
Surface tension of electrolytes at different temperatures with different LiF contents.

Similarly, this Figure 9 shows the change in the surface tension of the electrolyte with different contents of LiF at different temperatures. It can be seen that with the increase in LiF at 900 °C, the surface tension increased by more than 100%, and the highest reached 323. This indicated that the addition of LiF increased the surface tension at low temperatures. This phenomenon may be attributed to the addition of LiF, which greatly enhanced the strength and cohesion of the ion network inside the melt through its strong galvanic interaction [25]. The ions in the bulk phase were more tightly bound. They formed stronger and closer ion pairs with complex anions in the melt, resulting in a viscosity increase [26]. Then, the change in surface tension at 920–940 °C is similar to that of viscosity under the same conditions; the surface tension increases first and then decreases with the increase in LiF concentration. This phenomenon may occur because the content of LiF in the melt is small, and the addition of a small amount of LiF significantly enhances the strength of the ion network inside the melt, which is far more than the limited destructive effect of F on the network. Therefore, the surface tension increases with the increase in LiF. When the content is more than 3%, the excessive F^−^ ions will destroy the original large aluminum oxyfluoride complex ion group in the melt and make it dissociate into smaller and simpler ion units. These small ions are more surface active and may tend to accumulate on the surface, thereby reducing the surface tension. Also, heat will aggravate the thermal motion of ions, thus strengthening the effect of high LiF content on reducing surface tension [27].

**Figure 10:**
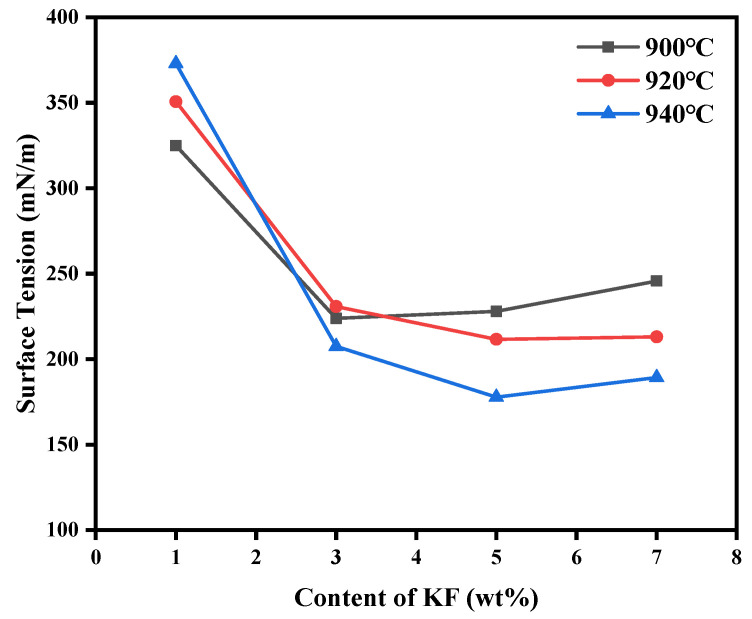
Surface tension of electrolytes at different temperatures with different KF contents when LiF content was 3 wt%.

In order to explore the synergism of LiF and KF on the surface tension of the electrolyte, the content of LiF was fixed, and the figure was obtained by changing the mass fraction of KF. It can be seen from the Figure 10 that all of the data in the experimental groups were higher than in the control group, indicating that the simultaneous addition of LiF and KF greatly improved the surface tension of the electrolyte [28]. At different temperatures, all curves showed a decreasing trend of surface tension with the increase in KF content. At the same time, increasing the temperature resulted in obvious surface tension change, and the slope of the curve increased. This phenomenon may be attributed to the fact that when the KF concentration was lower, a small amount of K^+^ had a weak interaction with complex anions in the melt (such as AlF_6_^3−^) due to its large ionic radius and low charge density. This made the K^+^ ions more inclined to migrate and accumulate on the surface of the melt rather than remain in the bulk phase. This surface enrichment behavior changed the composition and structure of the surface layer, effectively reducing the surface energy, and thereby reducing the surface tension. The addition of LiF usually enhanced the strength of the ionic network inside the melt and tended to increase the surface tension. With the addition of KF, its F^−^ ions may destroy the original large aluminum oxyfluoride complex ion group in the melt and make it dissociate into smaller and simpler ion units. These smaller ionic units may be more active on the surface. At the same time, the solvation behavior of K^+^ ions in the bulk phase may also change with the concentration. When the concentration of KF was high enough, its simplification and dilution effect on the melt structure dominated, thus jointly promoting the decrease in surface tension. The rising temperature aggravated the thermal motion of ions, making the melt structure more disordered. It also reduced the energy barrier of ion migration from the bulk phase to the surface. This phenomenon strengthened the surface adsorption process of K ions, making the effect of KF on reducing surface tension more significant [29].

## 4. Conclusions

In this work, we successfully explored the influence of different contents of LiF and KF on the viscosity and surface tension of the electrolyte at a cryolite ratio of three. The viscosity–content figure showed that the trends in the influence of the two regulators at different temperatures were similar; that is, the viscosity was reduced at both high LiF and high KF contents. The mechanisms of the two additives on the electrolyte were different. K^+^ ions increased the viscosity mainly by having a larger atomic volume than other ions in the electrolyte, but increasing the content of potassium ions provided more channels and reduced the viscosity. The phenomenon that lithium ions reduce viscosity is more attributed to their superior conductivity and lubricity. However, the impact on surface tension was different for the two of them. KF decreased the surface tension while LiF increased it. In conclusion, the research in this paper plays a guiding role in the subsequent production of aluminum electrolytes with a high content of LiF and KF.

## Data Availability

The original contributions presented in this study are included in the article. Further inquiries can be directed to the corresponding author.
